# A Prognostic Model for Triple-Negative Breast Cancer Patients Based on Node Status, Cathepsin-D and Ki-67 Index

**DOI:** 10.1371/journal.pone.0083081

**Published:** 2013-12-10

**Authors:** Liang Huang, Zhebin Liu, Sheng Chen, Yin Liu, Zhiming Shao

**Affiliations:** 1 Department of Breast Surgery, Fudan University Shanghai Cancer Center/Cancer Institute, Shanghai, P. R. China; 2 Department of Oncology, Shanghai Medical College, Fudan University, Shanghai, P. R. China; 3 Institutes of Biomedical Science, Fudan University, Shanghai, P. R. China; Yokohama City University School of Medicine, Japan

## Abstract

**Objective:**

The aim of this study was to evaluate clinicopathologic factors that could possibly affect the outcome of patients with triple negative breast cancer and subsequently build a prognostic model to predict patients’ outcome.

**Methods:**

We retrospectively analyzed clinicopathologic characteristics and outcome of 504 patients diagnosed with triple-negative invasive ductal breast cancer. 185 patients enrolled between 2000 and 2002 were designated to the training set. The variables that had statistically significant correlation with prognosis were combined to build a model. The prognostic value of the model was further validated in the separate validation set containing 319 patients enrolled between 2003 and 2006.

**Results:**

The median follow-up duration was 66 months. 174 patients experienced recurrence, and 111 patients died. Positivity for ≥4 lymph nodes, Cathepsin-D positivity, and Ki-67 index ≥20% were independent factors for DFS, while the lymph nodes status and Ki-67 index were the prognostic factors for OS. The prognostic model was established based on the sum of all three factors, where positivity for ≥4 lymph nodes, Cathepsin-D and Ki-67 index ≥20% would individually contribute 1 point to the risk score. The patients in the validation set were assigned to a low-risk group (0 and 1 point) and a high-risk group (2 and 3 points). The external validation analysis also demonstrated that our prognostic model provided the independent high predictive accuracy of recurrence.

**Conclusion:**

This model has a considerable clinical value in predicting recurrence, and will help clinicians to design an appropriate level of adjuvant treatment and schedule adequate appointments of surveillance visits.

## Introduction

Breast cancer remains the most frequently diagnosed cancer and the leading cause of cancer death in females worldwide, despite the improvement of early screening and adjuvant treatment. It is a heterogeneous disease highly variable with respect to its biology, etiology, and treatment options. Molecular pathological characterization and gene expression profiling are useful for identifying breast cancer subtypes with different clinical characteristics and developing therapeutic options[1]. DNA microarray analysis has classified tumors based on gene expression patterns and each distinct pattern correlates with prognostic markers of overall survival (OS) and disease-free survival (DFS). There are five different subtypes: luminal A and B, HER2-enriched, basal-like, and normal breast-like tumors[2]. Triple-negative breast cancer (TNBC), an aggressive variant of breast cancer characterized by lack of expression of the estrogen receptor (ER) , progesterone receptor (PR) and the human epidermal growth factor receptor 2 (HER-2) accounts for 15-26% of breast cancer[3,4]. 

A majority of TNBC is invasive ductal carcinoma of no special type, and the remaining is medullary carcinoma, invasive lobular, metaplastic carcinoma, etc. The triple negativity can occur in many histological subtypes of breast cancer, with possible implications on their pathogenesis, progression and prognosis[5,6]. On the other hand, most triple-negative tumors have pathobiological features in common with basal-like breast cancers. Basal-like breast tumors are preferentially low in ER and HER2 expression, and are significantly associated with several basal cytokeratin (CK) markers, including CK5/6, CK14, CK17, and the epidermal growth factor receptor (EGFR) [7]. A common misconception is that all basal-like breast cancers are TNBC; however, only 77% of basal-like breast cancers are triple-negative, with 71%–91% of TNBC being basal-like[8]. 

The breast cancer patients with higher histologic grade, larger size, high ERK protein expression, low E-cadherin expression and Ki-67 staining may have a tendency toward local and visceral metastases[3,9–11]. However, they are less predictive power for TNBC patients, despite that a large numbers of clinical and pathological factors have been studied to determine their value in predicting prognosis in patients who were diagnosed with TNBC. In addition, the Nottingham Prognostic Index (NPI) calculated by using tumor size, grade and lymph node score, is currently used for all types of breast cancer[12]. However, the value of the NPI was tested in fewer cohort of TNBC subgroup[13,14]. Thus, the prognostic model that can better predict the outcome of TNBC patients is clinically needed.

In this study, we accessed several clinical and pathologic characteristics of 185 TNBC patients in order to identify additional prognostic markers that can identify tumors with more aggressive behavior and develop a prognostic model comprised of the significant biomarkers. We then validated the model in additional 319 patients in the same institution and demonstrated that the predictive model is successfully to discriminate the high-risk group of TNBC patients. Our proposed model provides the potential value that could be used to tailor treatment and surveillance strategies.

## Methods

### Patients

A total of 504 eligible TNBC patients undertaking breast surgery between 2000 and 2006 at Shanghai Cancer Center were retrospectively analyzed. All patients had the following criteria in this study: (1) histologically confirmed mainly invasive ductal breast carcinoma, (2) a unilateral and non-inflammatory tumor, (3) status of ER, PR and HER-2 were available and negative, (4) patients had complete follow-up history, (5) the pathologic tissues were available for immunohistochemistry of other routine biomarkers in pathological department. Patient management was handled by the same department of surgeons, and the diagnosis was assessed by two senior pathologists. Conservative treatment and node resection along with radiotherapy, chemotherapy were applied according to current guidelines at that time, and all patients did not receive endocrine and trastuzumab treatment. The retrospective study was approved by the Ethics Committee of Shanghai Cancer Center. All patients gave their written informed consent before inclusion in this study.

### IHC analysis

Tissue samples were fixed in formalin and embedded in paraffin. Hematoxylin and eosin stained slide from each tissue was examined microscopically to confirm the presence of tumor before biomarker evaluation. Immunohistochemistry was performed on 4-μm-thick deparaffinized sections on plus-coated slides, and immunohistochemical staining was carried out according to the manufacturer’s instructions. Endogenous peroxidase and biotin were blocked with 3% hydrogen peroxide and the Avidin-Biotin blocking kit (Vector Laboratories; Burlingame, CA, USA), respectively. This was followed by incubation with primary antibodies for 60 min at room temperature, appropriate secondary antibodies (Dako Cytomation; Carpinteria, CA, USA), labeled streptavidin-horseradish-peroxidase (Dako Cytomation; Carpinteria, CA, USA), DAB+chromogen, and 0.2% osmium tetroxide (Sigma Chemicals, St Louis, MO), followed by counterstaining with light hematoxylin. Appropriate positive controls for each antibody and negative controls using species-matched immunoglobulin to replace the primary antibody were run with each batch. 

### Evaluation of IHC staining

Positive tumor cells were quantified by at least 1,000 cells and expressed as percentage. Samples were independently evaluated by two trained pathologist in the blind procedure without knowing the patients’ background and clinical outcome. The cut-off for ER and PR positivity was 1% tumor cell with positive nuclear staining. HER-2 positivity was defined as a complete membrane staining in >10% of tumor cells. It was then divided into a qualitative scale from 0 to 3+, according to the criteria of a Hercep Test. Scores 0 and 1 were negative, and scores 2 and 3 were positive[15]. The cut-off for Ki67 positivity was 20% tumor cells with positive nuclear staining. The cut-off for other markers was 10% tumor cells with positive staining, including nuclear staining for p53, TopoIIa, Cyclin D1 and p27, cytoplasmic staining for Cathepsin D, Bcl-2, Nm-23, BAX, MDR, GSTn and PS2. The following antibodies were used: ER (M7047, clone 1D5), PR (M3569, clone 636), HER-2 (A0485), Ki-67 (M7240, clone MIB-1), Cathepsin D ((polyclonal rabbit antibody), p53 (M7001, clone DO-7), Nm-23 (A096), Bcl-2 (M0887, clone 124), BAX (A3533), p27 (M7203, clone SX53G8), and Cyclin D1 (M3635,clone SP4) that were purchased from Dako, Denmark. TopoIIa (clone Ki-S1), MDR (MDR-1, clone 33A6), GSTn (clone 353-10), and PS2 (clone PS 2.1) that were obtained from Changdao, China.

### Statistical analysis

The chi-square test was used to compare clinicopathological parameters or expression levels of molecules in patients between the two sets. Descriptive statistics were calculated to summarize patient characteristics, tumor size, and biomarker levels of surgical tumor samples. Survival results were last updated in October 2012. Disease-free survival (DFS) was defined as the elapsed time between the date of the first diagnosis and the date of the first relapse (local recurrence and distant metastasis). Overall survival (OS) was defined as the length of time from the date of operation to death. Patients without events or death were censored at the last follow-up. Survival curves were established according to the Kaplan–Meier method. The log-rank test was used for univariate comparisons of survival endpoints. The Cox regression procedure was carried out to assess the relative influence of prognostic factors on DFS and OS. All tests were considered significant at two-sided P < 0.05. All analyses were carried out using SPSS 17.0 (SPSS, Chicago, IL, USA)

## Results

### Patient characteristics

The median age of 504 women with TNBC was 51 years old (rang 23-85 years). All patients had received a curative operation of a mastectomy or a conservative surgery with axillary lymph node dissection in Shanghai Cancer Center between 2000 and 2006. 185 patients enrolled between 2000 and 2002 were included in the training set, while 319 patients recruited between 2003 and 2006 were designed in the validation set. The clinicopathological features of patients in the training set and validation set were shown in Table 1, which were well balanced. Approximately 38% patients had positive lymph nodes. There were 47 and 93 patients in T1 stage (tumor size ≤ 2 cm), 121 and 189 patients in T2 stage (tumor size 2 to 5 cm), 17 and 37 patients in T3 (tumor size ≥ 5 cm) in the training and validation sets, respectively. In addition, 131 and 230 patients were histological grade I /II, and 54 and 89 patients were grade III, in both sets, respectively. 475 patients (94.3%) received the cytotoxic chemotherapy after surgery. In training set and validation set, 142 patients and 177 patients with anthracycline based regimen, 31 patients and 30 patients with cyclophosphamide, methotrexate and 5-fluorouracile (CMF)-based chemotherapy. 95 patients in validation set with taxol based regimen.

**Table 1:** Clinicopathological characteristics of patients in the training and validation sets.

	Training set		Validation set		
Variables	No. of patients (%)		No. of patients (%)		P value
Age					
Median	50		52		
Range	28-80		23-85		
Menopausal status					
Pre	102	(55.1)	165	(51.7)	0.460
Post	83	(44.9)	154	(48.3)	
Tumor stage					
T1	47	(25.4)	93	(29.2)	0.377
T2	121	(65.4)	189	(59.2)	
T3	17	(9.2)	37	(11.6)	
Lymph nodes status					
Positive	73	(39.5)	119	(37.3)	0.631
Negative	112	(60.5)	200	(62.7)	
Histologic grade					
I/II	131	(70.8)	230	(72.1)	0.757
III	54	(29.2)	89	(27.9)	
Ki-67 index					
Low group	92	(49.7)	142	(44.5)	0.258
High group	93	(50.3)	177	(55,5)	
Cathepsin-D					
Low group	45	(24.3)	91	(28.5)	0.306
High group	140	(75.7)	228	(71.5)	

### Predictors for Survival of the training set

The median follow-up duration for the patients in the training set was 69.7 months; among them, 69 patients (37.3%) experienced a local or distant recurrence, and 40 patients (21.6%) died. We analyzed clinicopathologic characteristics of the patients using the Cox univariate survival analysis to identify potential predictors for DFS and OS (Table 2). The univariate analysis showed that the lymph node status (P<0.001), Cathepsin-D (P=0.024), and Ki-67 index (P=0.032) were prognostic factors for DFS. The multivariate analysis further demonstrated that the lymph node status (HR: 3.69, 95% CI: 2.23-6.11, P<0.001), Cathepsin-D (HR: 2.12, 95% CI: 1.05-4.28, P=0.035) and Ki-67 index (HR: 1.74, 95% CI: 1.07-2.84, P=0.025) remained to be independent prognostic factors , while menopausal status, tumor stage and histologic grade did not have statistical significance. By contrast, lymph node status (P<0.001) and Ki-67 (P=0.044) were associated with OS by the univariate analysis. The multivariate analysis indicated that the lymph node status (HR: 4.10, 95% CI: 2.17-7.75, P<0.001) and Ki-67 (HR: 2.01, 95% CI: 1.05-3.83, P=0.034) were still significant predictors for OS.

**Table 2:** The univariate analysis of DFS and OS for patients of the training set.

	DFS				OS			
Variables	P value	HR	95% CI		P value	HR	95% CI	
Menopausal status								
Pre vs. Post	0.352	1.253	0.780-2.012		0.446	1.273	0.685-2.366	
Tumor stage								
T1 vs. T2/T3	0.609	1.154	0.667-1.995		0.069	2.239	0.940-5.334	
Lymph nodes status								
<4 vs. ≥4 nodes	<0.001	3.483	2.141-5.665		<0.001	4.072	2.188-7.579	
Histologic grade								
I/II vs. III	0.898	1.035	0.614-1.743		0.182	1.548	0.815-2.938	
Cathepsin-D								
Neg vs. Pos	0.024	2.162	1.104-4.231		0.068	2.393	0.937-6.109	
p53								
Neg vs. Pos	0.102	0.652	0.390-1.089		0.140	0.593	0.296-1.188	
TopoIIa								
Neg vs. Pos	0.676	1.127	0.644-1.972		0.253	1.609	0.711-3.639	
Nm23								
Neg vs. Pos	0.762	0.928	0.572-1.505		0.376	1.357	0.690-2.670	
Bcl-2								
Neg vs. Pos	0.510	0.852	0.529-1.372		0.353	0.741	0.393-1.396	
BAX								
Neg vs. Pos	0.765	1.076	0.666-1.737		0.343	1.364	0.718-2.592	
MDR								
Neg vs. Pos	0.674	1.107	0.689-1.780		0.563	1.203	0.643-2.252	
GSTn								
Neg vs. Pos	0.396	0.763	0.409-1.424		0.659	1.235	0.483-3.157	
PS2								
Neg vs. Pos	0.443	1.204	0.749-1.936		0.233	1.470	0.780-2.768	
p27								
Neg vs. Pos	0.711	0.913	0.562-1.481		0.792	0.917	0.484-1.741	
Cyclin D1								
Neg vs. Pos	0.976	0.993	0.614-1.606		0.810	1.080	0.577-2.023	
Ki-67 index								
Neg vs. Pos	0.032	1.693	1.046-2.741		0.044	1.935	1.018-3.677	

DFS: disease-free survival; OS: overall survival; HR: hazard ratio; CI: confidence interval; Neg: negative; Pos: positive.

### Prognostic model

**Figure 1:**
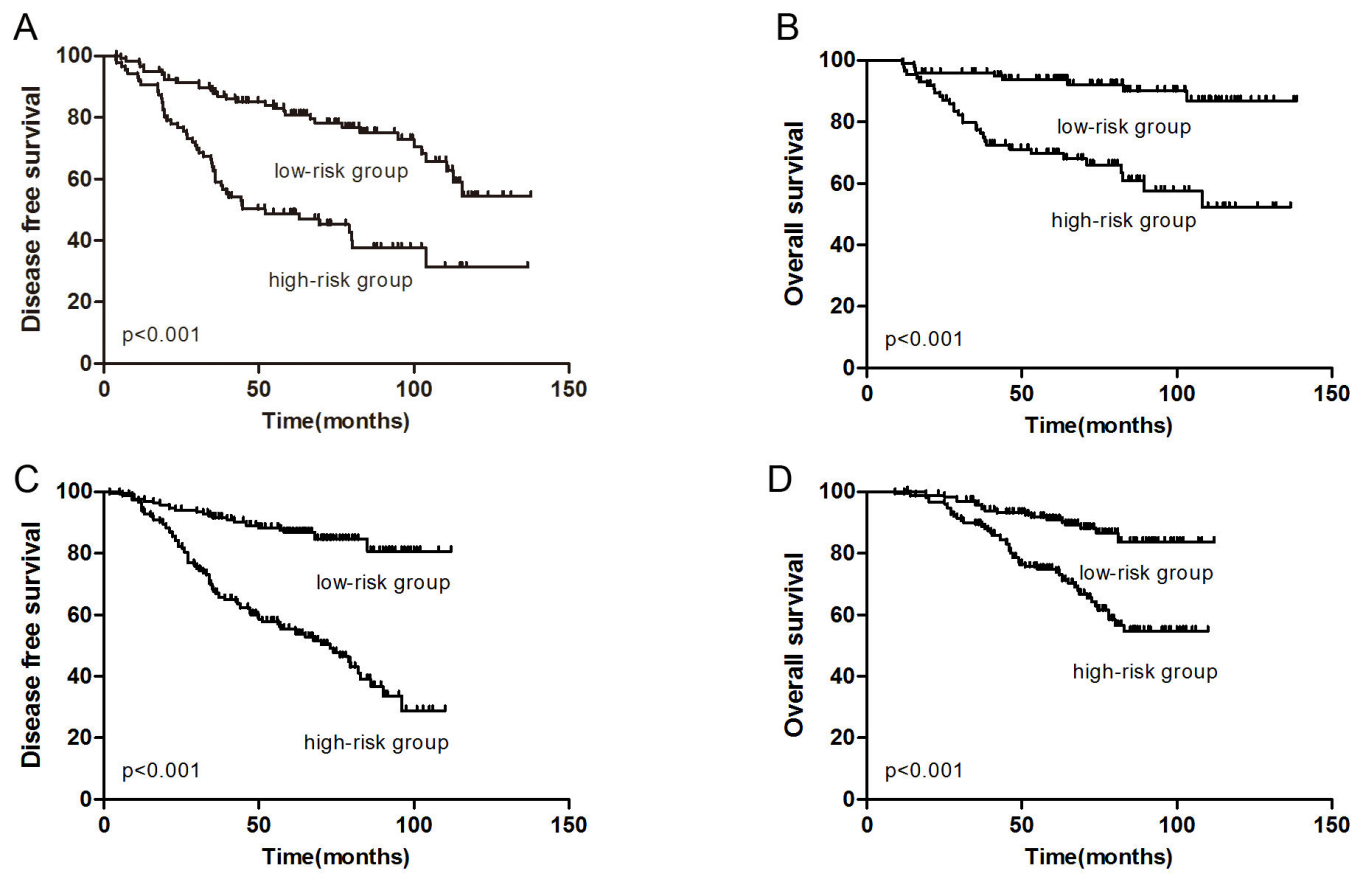
Kaplan-Meier curves for DFS (A) and OS (B) of TNBC patients in the training set according to the prognostic model. Kaplan-Meier curves for DFS (C) and OS (D) in the validation set.

Because the lymph node status, Cathepsin-D and Ki-67 index were factors strongly associated with outcome of TNBC patients, we built up a prognostic model based on the summation of points scored by the features of these three factors in tumor tissues. For example, positive for ≥4 lymph nodes, positive for Cathepsin-D expression or Ki-67 index ≥20% would be individually recorded 1 point to the prognostic score. According to the prognostic score, patients were assigned to eight categories (Table 3). Based on the prognostic score, patients in the low-risk group (0 and 1 point) and the high-risk group (2 and 3 points) had significant different outcome (HR=4.03, P<0.001 for DFS; HR=4.87, P<0.001 for OS) (Figure 1A and Figure 1B). The 5-year DFS rates in low-risk group and high-risk group were 85.9% and 50.0%, respectively (P<0.001), while the 5-year OS rates were 93.9% and 73.3%, respectively (P<0.001). The multivariate analysis showed that the combination of ≥4 lymph nodes Cathepsin-D positivity and high Ki-67 index was a more significant prognostic indicator than either factor alone. 

**Table 3:** Prognostic model comprised of risk categories based on the status of predictors.

	Number of patients	Status of predictors						
category		≥4+ nodes	Cathepsin-D	Ki-67≥20%	Risk points	Risk group	HR for DFS	HR for OS
1	14	-	-	-	0	Low risk	1.0	1.0
2	6	+	-	-	1	Low risk		
3	57	-	+	-	1	Low risk		
4	23	-	-	+	1	Low risk		
5	15	+	+	-	2	High risk	4.029	4.866
6	2	+	-	+	2	High risk	(2.379-6.824)	(2.311-10.247)
7	50	-	+	+	2	High risk		
8	18	+	+	+	3	High risk		

### External validation of the model

In order to further validate the proposed prognostic model which is able to distinguish the TNBC patients with different outcomes, we tested the model using the survival data from the TNBC patients in the validation set. Table 1 showed that 37.3% patients were lymph nodes positive; 71.5% patients expressed Cathepsin-D; 55.5% patients had high Ki-67 index. According the prognosis score, 153 patients belonged to the high risk group, which had higher recurrence rate (52.9%) than the low risk group (14.5%) (HR: 4.50, 95% CI: 2.85-7.10, P<0.001) (Figure 1C). The overall survival rate of the high-risk group was worse than those of the low-risk group (HR: 3.28, 95% CI: 1.94-5.54, P<0.001) (Figure 1D). The multivariate cox analysis demonstrated that the prognostic model remained to be the independent predictive factor in training set and validation set. Receiver operating characteristic curve (ROC) analysis for recurrence showed an AUC of 0.696 (95% CI: 0.616–0.775) for the training set and 0.717 (95% CI: 0.658–0.777) for the validation set. In validation set, positive predictive value is 57.0%, and negative predictive value is 79.8%.

## Discussion

In this study, we evaluated the relationships between the clinical outcomes, clnicopathological characterics, and expression levels of prognostic biomarkers in patients with confirmed triple-negative breast cancer. Our analysis demonstrated that the combination of 3 prognostic factors, including positive for ≥4 lymph nodes , high Cathepsin-D expression and high Ki-67 index, would provide a strong prognostic power to differentiate the patients with worse outcome. Based on the score system consisted of local or distant metastasis in multiple lymph nodes, elevated Cathepsin-D expression and high Ki-67 proliferation index , we built up a mathematic prognostic model to distinguish the TNBC patients with the low survival rate (high-risk group) and the high survival rate (low-risk group). The proposed model demonstrates that the high-risk group has worse outcomethan the low-risk group in both training and validation sets. Our model would help select the subgroup of patients with high prevalence of local recurrence or distant metastasis. The cost-benefit ration and the selection of the most efficient design can be determined on the basis of the population selected using the model.

Despite the fact that Cathepsin-D plays a critical role in a non-specific protein degradation in a markedly acidic environment of lysosomes, overexpression of Cathepsin-D in breast tumors is associated with the increased metastatic potential and the poor survival rate, in part by facilitaing cancer cell proliferation, fibroblast outgrowth, angiogenesis and metastasis. A recent study found that overexpression of Cathepsin-D in neuroblastoma cells attenuates doxorubicin-induced apoptosis through the activation of both the Akt-dependent pro-survival and the Bcl-2-dependent anti-apoptotic pathways[16], suggesting that elevated Cathepsin-D expression in tumors may protect cancer cells from chemotherapy. Indeed, a large-scale study of 2,810 lymph node-negative breast cancer patients revealed that patients with a high or moderate expression level of Cathepsin-D in a primary tumor have a poor prognosis , independent of histologic grade, hormone receptor status, or tumor size[17]. In addition, Cathepsin-D expression level is not associated with number of lymph nodes positive for metastasis, the histological grade and the dimension of the tumor[18]. Consistent with these observations, the multivariate analysis in our study showed that Cathepsin-D expression remains to be an independent prognostic factor for DFS of the TNBC patients. However, its prognostic values for OS decreases as the lymph node status is the only factor associated with the overall survival rate of patients. This data supports the finding in previous studies that Cathepsin-D expression is not correlated with survival in either lymph node-negative or -positive patients[19,20]. 

Ki-67 is a non-histone nuclear protein that is closely linked to proliferation cells. High Ki-67 expression is associated with a higher histologic grade, larger tumor size, the presence of axillary lymph nodal metastasis, and worse outcome[21–23]. In neoadjuvant chemotherapy (NAC), higher Ki-67 expression is associated with better pathologic complete response (pCR); however, it is a strong prognostic factor for worse survival of patients who fail to achieve pCR after NAC[24–26], suggesting that Ki-67 can be used for classification of patients with different responses and prognosis. Ki-67 is one of the markers for chemosensitivity in breast carcinomas, but the correlation between Ki-67 expression and chemosensitivity in the triple-negative phenotype is not clear[10], probably due to heterogeneous characteristics of TNBC. Furthermore, standardized staining methods and image analysis for making a consistent cut-off value for Ki-67 may be required to achieve a comparative measurement[10,27]. Our study indicates that a cut-off level of 20% for Ki-67 index is adequate to demonstrate a correlation with recurrence.

The size of the primary tumor and the number of positive lymph nodes have an inverse relationship with prognosis and survival in breast cancer. The sixth edition of staging in breast cancer has included the nodal status as the most important prognostic factor. In large retrospective analysis, TNBC patients with N0/1 have better DFS than those with N2/3 (68% vs. 43%, P<0.001) [28]. On contrary, the prognostic value of other biomarkers, such as p53, is ambiguous. The previous studies found that p53 status was a specific prognostic factor in TNBC patients treated with an adjuvant anthracycline-based regimen or in the subgroup younger than 50 years, although the sample size was small[29,30]. Some studies showed that p53 status was not a specific prognostic factor in TNBC patients treated by adjuvant chemotherapy[10]. Our analysis data showed that high p53 expression had a moderate association with a good outcome (P=0.102). It is conceivable that p53 mutation may provide a more important value in predicting patient prognosis than p53 expression level alone[31]. 

Currently, the factors associated with the poor clinical outcomes in TNBC patients have been studied using gene expression profiles and more advanced genomic techniques. However, these approaches are not widely available. The application of therapeutic options and the assessment of the accompanied prognosis are dependent on the common tumor-related characteristics in patients. Based on the nodal status, Cathepsin-D expression and Ki-67 index in a group of patients as a training set, we built a faithful model that had considerable clinical value in predicting recurrence in another group of TNBC patients. This prognostic model can help clinicians to provide TNBC patients with appropriate adjuvant treatments and surveillance visits.
